# In-Cabin Air Quality during Driving and Engine Idling in Air-Conditioned Private Vehicles in Hong Kong

**DOI:** 10.3390/ijerph15040611

**Published:** 2018-03-27

**Authors:** Natasha Maria Barnes, Tsz Wai Ng, Kwok Keung Ma, Ka Man Lai

**Affiliations:** Department of Biology, Hong Kong Baptist University, Kowloon Tong, Hong Kong, China; natashamaria41@gmail.com (N.M.B.); wai64@yahoo.com.hk (T.W.N.); kkma@hkbu.edu.hk (K.K.M.)

**Keywords:** in-cabin air quality, air-conditioned vehicles, idle engine, volatile organic compounds (VOCs)

## Abstract

Many people spend lengthy periods each day in enclosed vehicles in Hong Kong. However, comparably limited data is available about in-cabin air quality in air-conditioned private vehicles, and the car usage that may affect the air quality. Fifty-one vehicles were tested for particulate matter (PM_0.3_ and PM_2.5_), total volatile organic compounds (TVOCs), carbon monoxide (CO), carbon dioxide (CO_2_), airborne bacteria, and fungi levels during their routine travel journey. Ten of these vehicles were further examined for PM_0.3_, PM_2.5_, TVOCs, CO, and CO_2_ during engine idling. In general, during driving PM_2.5_ levels in-cabin reduced overtime, but not PM_0.3_. For TVOCs, 24% vehicles exceeded the recommended Indoor Air Quality (IAQ) level in offices and public places set by the Hong Kong Environmental Protection Department. The total volatile organic compounds (TVOC) concentration positively correlated with the age of the vehicle. Carbon monoxide (CO) levels in all of the vehicles were lower than the IAQ recommendation, while 96% vehicles exceeded the recommended CO_2_ level of 1000 ppmv; 16% vehicles >5000 ppmv. Microbial counts were relatively low. TVOCs levels at idle engine were higher than that during driving. Although the time we spend in vehicles is short, the potential exposure to high levels of pollutants should not be overlooked.

## 1. Introduction

With people in modern societies spending as much as 70% of their time indoors, it is not surprising that factors contributing to poor Indoor Air Quality (IAQ) are receiving significant attention from researchers, government officials, and the general public. However, despite the fact that many people spend upwards of an hour each day inside enclosed vehicles in industrialized countries, such as the United States or those in the European Union [1], and progressively more car-use air-cleaning devices are produced and sold, comparably limited data is available about in-cabin air quality, as opposed to that in offices and other indoor places. In Hong Kong, vehicles are commonly air-conditioned to maintain temperature comfort and to reduce outdoor polluted air from entering the cabin. Studies have reported a higher level of exposure to particulate matter (PM) and total volatile organic compounds (TVOCs) inside the vehicle cabin as compared to the ambient environment, because the outside air pollutants can accumulate in the car cabin when the windows are open [2,3,4,5,6,7]. In addition to the outside environment, the interior environment can also affect the in-cabin air quality. An investigation which looked at 101 new Japanese private-use cars found that a total of 275 organic compounds were detected in the in-cabin microenvironment associated with the interior materials [8]. Specific carcinogenic organic compounds, e.g., benzene, toluene and formaldehyde, and other toxic gases e.g., carbon monoxide (CO) and nitrogen oxide (NOx), as well as residual tobacco smoke are also of concern [2,3,4,5,6,7]. In-cabin air quality is not only influenced by chemical pollution, but also by biological contamination via bacteria and fungi and their by-products e.g., endotoxins from bacteria and beta (1,3) glucans from fungi. Microbial odors are one of the most notable problems that car occupants can sense immediately [1,4,9,10]. Many of these pollutants are especially hazardous to people with respiratory problems or heart diseases [11,12,13]. Other than the outdoor environment and the car interior, car age and maintenance, the use of different cleaning and air freshener chemicals, as well as car usage, such as opening or closing windows and using different ventilation modes can affect the in-cabin air quality [3].

In Hong Kong, different guidelines and schemes are in place to improve and increase the public awareness and the regulation of outdoor air pollution, IAQ in public transport, car emission control, and indoor air quality in offices and public places that are mechanically ventilated. Still not many guidelines are available on the in-vehicle air quality [14,15,16,17,18,19,20,21,22]. For instance, the statutory ban against idling of motor vehicle engines was passed in 2011, prohibiting continuous engine idling for more than three minutes in any 60-min stationary period and aiming at controlling the impact of air pollution, heat and noise nuisance that is caused by engine idling on the pedestrians and shops along the roadside [23]. There is limited data available on the current state of in-cabin air quality in private vehicles in Hong Kong during driving and engine idling, and no guideline has been set on the levels of pollutants in vehicle cabins.

Vehicles are usually air-conditioned in hot and/or humid seasons in Hong Kong for temperature comfort. Moreover, due to the concern over outdoor air pollution, certain people may operate air-conditioners all-year-round at recirculation mode, making this enclosed environment a common indoor landscape in vehicles. Some commuters assume that the in-cabin air quality can be improved once the vehicle is air-conditioned, and once the windows and ventilation are tightly closed to separate the outside polluted air from the inside. Moreover, the in-vehicle car filter purports to reduce the in-cabin pollution level. Therefore, the present study aims to answer the question—what is the in-cabin air quality during driving and engine idling in air-conditioned private vehicles in Hong Kong? Collecting in-cabin air quality data can give an insight into the commuters’ exposure levels to different pollutants during their normal travel journey and to justify what mitigation measures and specifications of air-cleaning devices could improve the air quality.

## 2. Materials and Methods

### 2.1. Vehicle Recruitment and In-Cabin Air Measurements

Fifty-one vehicles were recruited and tested in this study on a voluntary and random basis from May to September 2017. This period was summer in Hong Kong and all of the vehicles were air-conditioned during the air measurements. Since some people may perceive that air-conditioned environments mean improved air quality with better temperature comfort, this cabin environment was selected to examine whether the air quality in cabin is comparable to a Good or Excellent Class of IAQ in offices and public places in Hong Kong that are set by the Hong Kong Environmental Protection Department (HKEPD) (Table 1); there are no guidelines on IAQ in private vehicles in Hong Kong. In-cabin air quality is influenced by both, inside and outside environments, as well as the car usage. The current study design measured the total outcomes of environmental concentration of different pollutants. During the study, a researcher entered the vehicle and measured the air quality in the backseat diagonally opposite to the side of the driver (to avoid interfering the driver and to allow for more direct airflow to the sampler). The driving route and time was determined by the driver, who was mostly driving from home to work or vice versa. The journey time was usually around 30 min. Fine and ultra-fine particulate matter with diameter 2.5 μm (PM_2.5_) and 0.3 μm (PM_0.3_), TVOCs, CO, CO_2_, temperature, and relative humidity (RH) were measured at one minute intervals from the start to the end of the journey. The total airborne bacteria and fungi were measured after 10 min of driving. Ten of the above 51 vehicles were selected randomly to conduct a more detailed investigation on PM_0.3_, PM_2.5_, TVOCs, CO, and CO_2_ during engine idling. At idle engine, after the car engine was turned on for 10 min, the aforementioned parameters were measured for 10 min, as indicated above. The reason for this sampling lag time was based on the TVOCs measurement in our preliminary studies, which showed that in most cases TVOCs levels increased gradually and became more stable after 10 min of engine operation. Collecting air samples after the lag time helped in determining the TVOCs at their relatively equilibrium levels in the cabin.

### 2.2. IAQ Measurements

PM_2.5_ and PM_0.3_ were measured using a TSI AeroTrak^®^ Handheld Particle Counter Model 9303 (TSI Inc., Shoreview, MN, USA). Particle count numbers (PtL^−1^) rather than particle weight counts were used in the measurement in order to examine the level of particles in-cabin and the change in these particle numbers based on two different sizes (0.3 and 2.5 micron), because particle size could affect their removal by car filters and the other processes in the cabin. TVOCs levels were measured using a Handheld VOC Monitor ppb RAE 3000 (RAE system Ltd., Hong Kong, China). CO and CO_2_ were measured using a TSI IAQ-Calc™ Indoor Air Quality Meter 7545. Total airborne bacterial and fungal counts were determined with a Portable Air Microbiological Sampler (pbi International) (total air sampled, 500 L) on Tryptic Soy Agar (TSA) and Potato Dextrose Agar (PDA), respectively. After incubation at 30 °C for 3 days, the bacterial and fungal colonies on the agar plates were counted. The temperature and RH inside the cabin were monitored using a HOBO temp/RH Logger UX100-003.

### 2.3. Questionnaires

Information about the vehicles, their usage and the driver’s perception of the outdoor, and in-cabin air quality was collected using a questionnaire and then grouped by the researcher to show the percentage distribution of different responses (Table 2).

### 2.4. Data Analysis

During the first 5 min of testing, some parameters fluctuated significantly, which may be due to the start of the car engine and air-conditioner, driver and researcher movement, and opening and closing the door. Therefore, these data were discarded, and the subsequent data (next 5 min) were averaged and analyzed. Total bacterial and fungal counts (collected during driving) in each vehicle were analyzed, too. The overall average of each parameter from all the vehicles were calculated.

To further interpret the data from these vehicles under different situations, the difference between the means and the correlation between different parameters, car conditions, and the driver’s perception of the air quality were performed using independent grouped *T*-test and Pearson Correlation model (e.g., in car age, mileage, usage and cleaning frequency analysis, individual values collected from the vehicles rather than the percentage in different groups were used in the analysis) (Statistical Package for Social Scientists, SPSS, SPSS Inc., Chicago, IL, USA), respectively.

## 3. Results

### 3.1. Overview of the Questionnaire Result

A majority of the vehicles tested (>80%) were within 10 years old and the mileage was less than 90,000 km (Table 2). Most of these vehicles were used regularly. More than half of these vehicles were cleaned at least every two months, but about 14% were rarely or never cleaned. Interestingly, although most of the respondents (86%) were aware of cleaning their vehicles, over 70% of the respondents were not aware of car filter maintenance. Since TVOCs were measured in this study, we asked the respondents whether any suspected agents that could contribute to in-cabin TVOCs were present in the vehicle, and if so, these items were removed during the test. Most of the vehicles (69%) were reported to have no TVOC related items. For the vehicles that had fragrance or alcohol gel in the cabin, these vehicles were ventilated until a relatively stable TVOC level was displayed before testing. The air-conditioning system was in-use in all of the vehicles during this study. However, nearly 65% of the respondents operated the air-conditioning system all year round as a way to improve the air quality. About 73% of the vehicles used the recirculation mode of ventilation. Most of the respondents perceived the outdoor air quality as “average”. Most of the vehicles (70%) had no reported odor problems, and only about 6% of the respondents had smoked in the vehicle. After further data analysis, some of this information (ventilation modes, car age, and mileages) was used to correlate with different IAQ parameters, and the results showing a significant correlation are reported below.

### 3.2. PM_2.5_ and PM_0.3_

**Figure 1:**
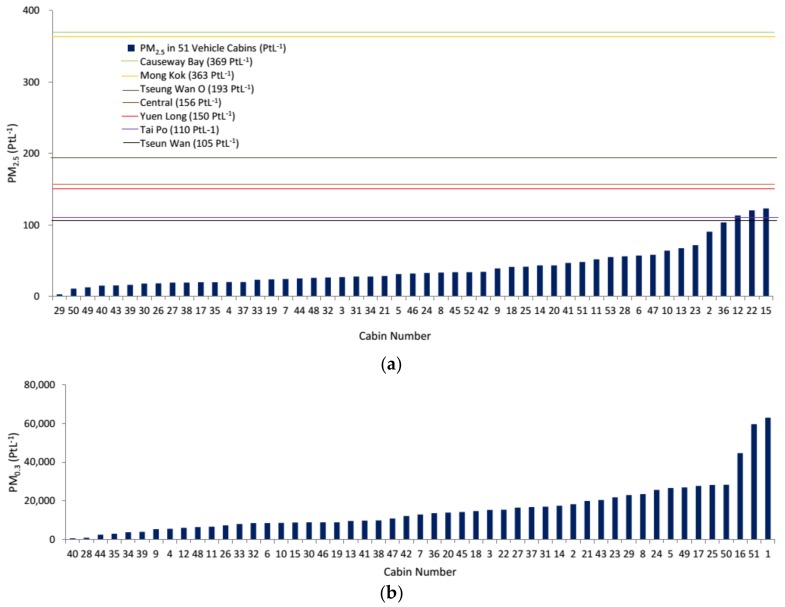

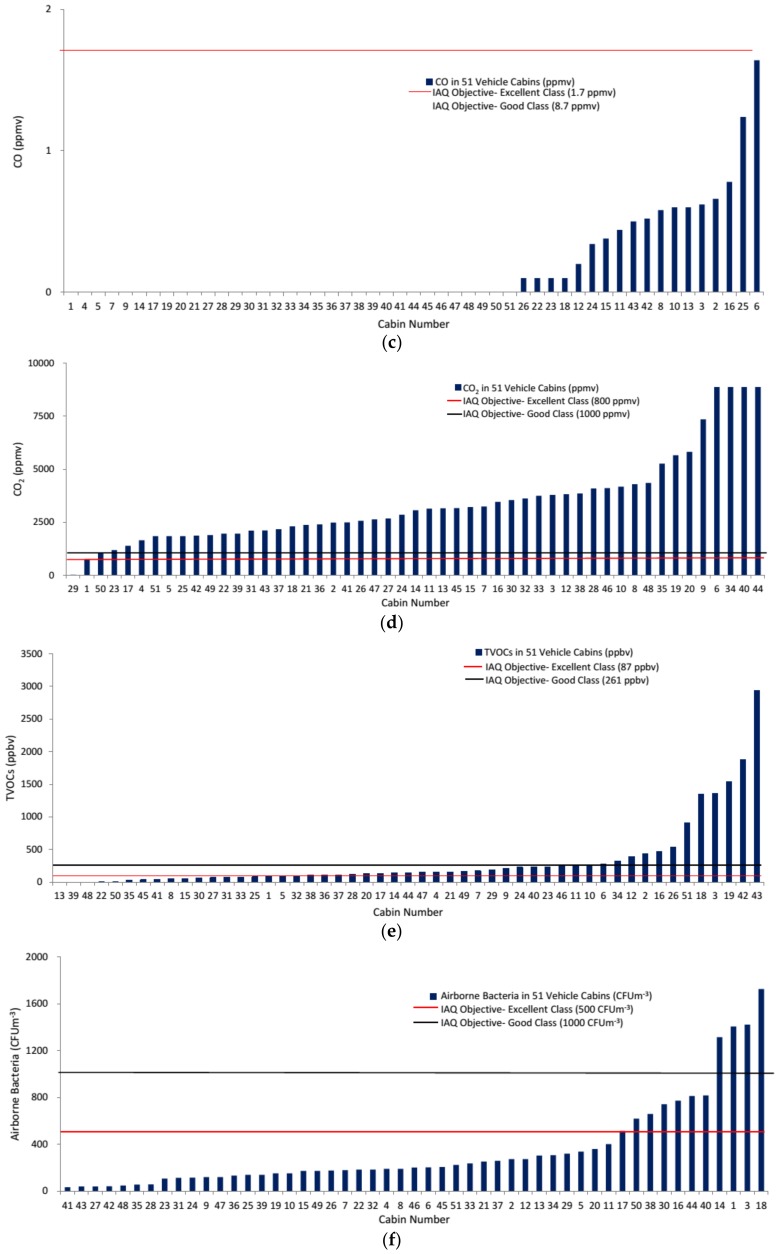

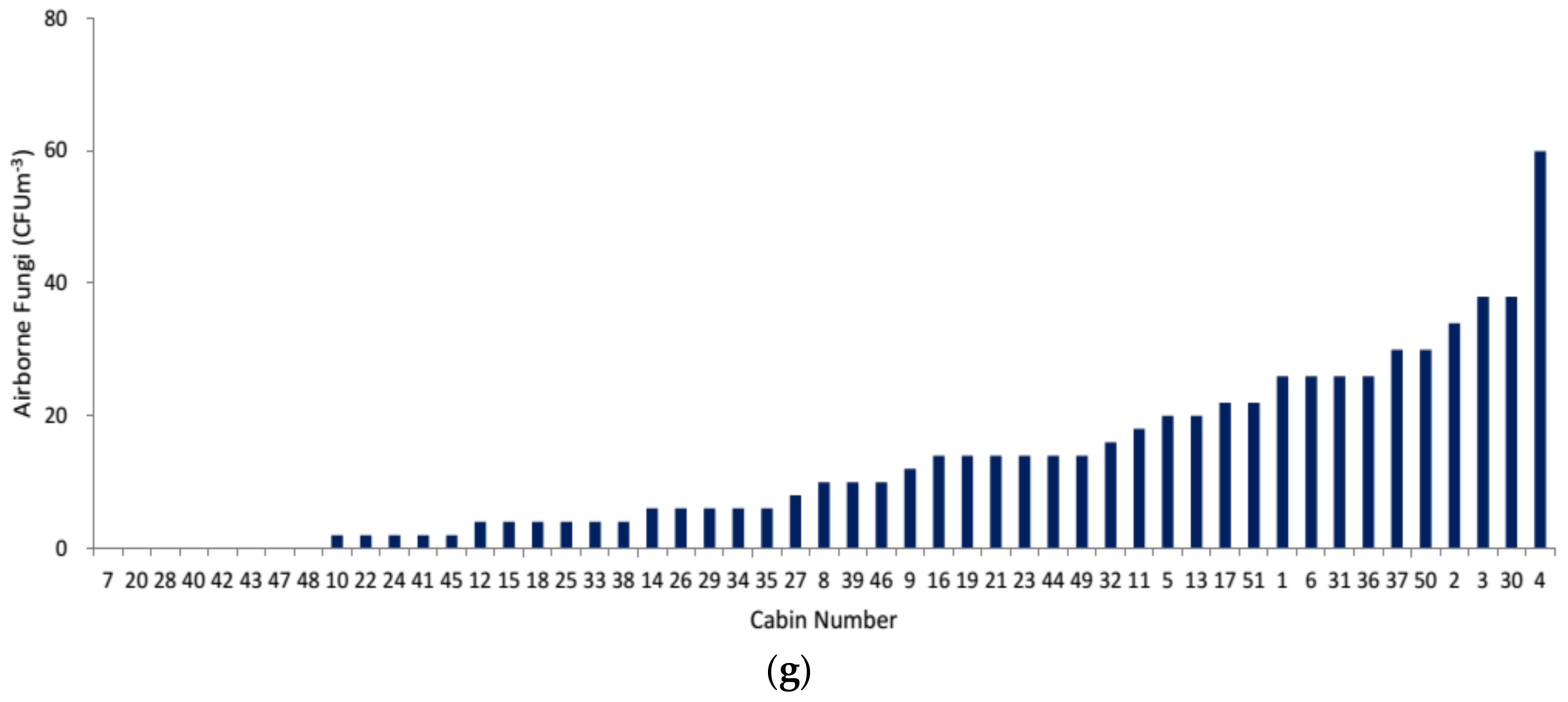
The Indoor Air Quality (IAQ) parameters in the in-cabin microenvironment during driving, particulate matter (PM)_2.5_ (**a**); PM_0.3_ (**b**); carbon monoxide (CO) (**c**); CO_2_ (**d**); total volatile organic compounds (TVOCs) (**e**); Airborne Bacteria (**f**); and, Airborne Fungi (**g**). As there are no IAQ guidelines for PM_0.3_ and PM_2.5_, the PM_2.5_ levels were compared with the levels recorded in different districts in Hong Kong.

**Figure 2:**
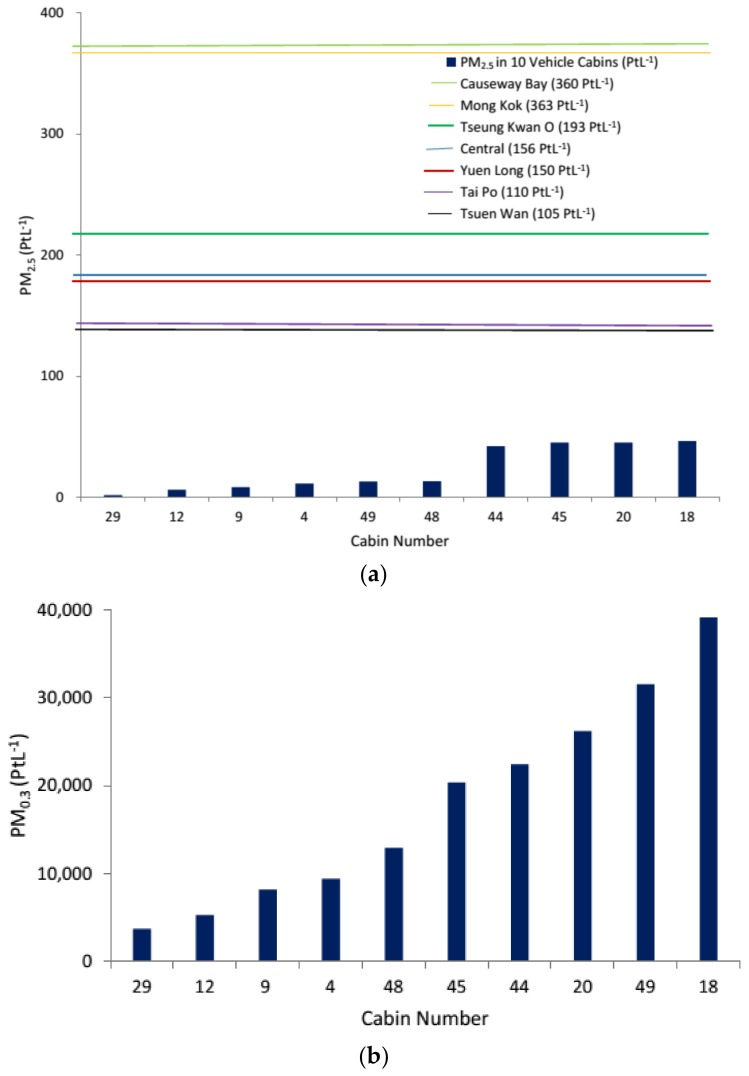

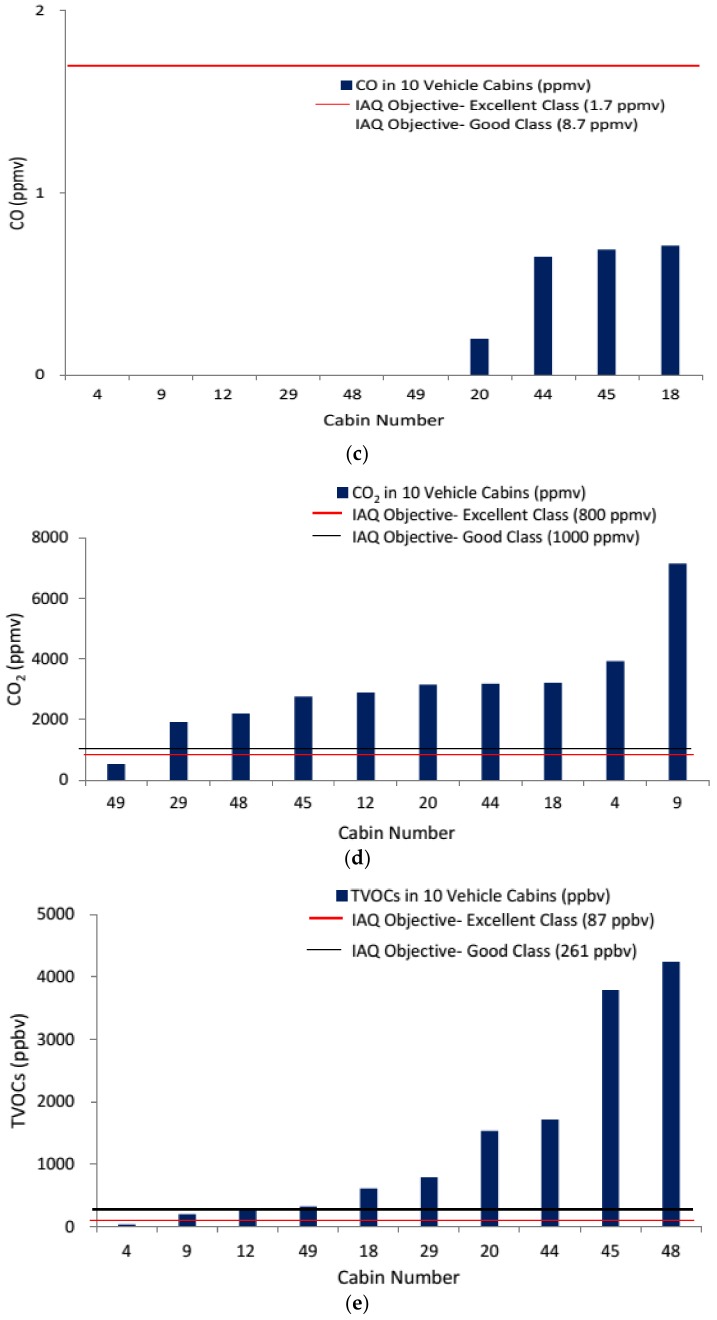
The IAQ parameters in the in-cabin microenvironment during engine idling, PM_2.5_ (**a**); PM_0.3_ (**b**); CO (**c**); CO_2_ (**d**); and, TVOCs (**e**). As there are no IAQ guidelines for PM_0.3_ and PM_2.5_, the PM_2.5_ levels were compared with the levels recorded in different districts in Hong Kong.

PM_2.5_ is one of the parameters commonly monitored in outdoor air as it is closely linked to the combustion process, such as from car exhaust. In this study, the mean PM_2.5_ (driving) was 40 ± 28 PtL^−1^ and was significantly higher than 23 ± 19 PtL^−1^ at idle engine (Figure 1a and Figure 2a). As compared to the roadside PM_2.5_ measurements in different districts (highest 369 PtL^−1^ in Causeway Bay to lowest 105 PtL^−1^ in Tsuen Wan), the levels of in-cabin PM_2.5_ that were recorded in this study were mostly lower than the outdoor levels. We noticed that PM_2.5_ decreased gradually in-cabin over time. An average reduction of 34% in 10 min was obtained among the tested vehicles. PM_0.3_ was measured to compare with the trend that was observed with PM_2.5_. Interestingly, the mean PM_0.3_ count during driving and engine idling were not significantly different, and no trend was observed (Figure 1b and Figure 2b). In general, a greater variation in PM was recorded during driving when compared to engine idling.

### 3.3. CO

CO was detected in 35% of the vehicles during driving and 40% of the vehicles during engine idling. The mean CO concentrations during these conditions were not significantly different and within the IAQ recommended Excellent Class (1.7 ppmv) (Figure 1c and Figure 2c).

### 3.4. CO_2_

Among the vehicles that were tested, 96% exceeded the IAQ recommendation on CO_2_ (Good Class, <1000 ppmv) during driving (mean—3413 ppmv; four vehicles exceeded the maximum detection limit, 8888 ppmv, and this value was used in the calculation of the mean) and 90% exceeded the Good Class level during engine idling (mean—3096 ppmv) (Figure 1d and Figure 2d). Alarmingly, eight vehicles (almost 16%) had CO_2_ levels over 5000 ppmv (five times that of the recommendation) during driving condition.

### 3.5. TVOCs

During driving, 24% of the vehicles did not achieve Good or Excellent Class levels for TVOCs. At idle engine condition, 70% of the vehicles were of concern. The mean TVOC concentration at idle engine (1351 ppbv) was significantly higher than that during driving (331 ppbv) (Figure 1e and Figure 2e). Both of the mean values were considerably higher than the IAQ recommendation of 261 ppbv. A significant positive correlation (0.319, *p* < 0.05) between TVOCs and car age was obtained. Most vehicles (69%) had not used any agent that could contribute to the in-cabin TVOCs (Table 2).

### 3.6. Microbial Counts

Only three vehicles had bacterial counts above 1000 CFUm^−3^ (mean = 350 CFUm^−3^) (Figure 1f). The mean fungal count was also low (13 CFUm^−3^) (Figure 1g). Additionally, a significant positive correlation (0.30, *p* < 0.05) was observed between PM_2.5_ and bacterial counts.

## 4. Discussion

### 4.1. Impacts of High CO_2_ Concentrations on Air Quality, Comfort, Health and Road-Safety

CO_2_ and TVOCs could be present in-cabin at an alarming level as compared to the HKEPD guideline set for offices and public places in Hong Kong. The guideline applies to an 8-h exposure, but a high pollutant level present in vehicles, albeit during a brief exposure, may cause an IAQ and health concern. We identified two potential high-risk situations 1. CO_2_ during driving and 2. TVOCs during engine idling.

In IAQ assessment, Good Class CO_2_ helps indicate an adequate ventilation level to maintain air comfort. Jung (2013) modeled the CO_2_ concentration in vehicle cabins with CO_2_ emission from occupants [24]. CO_2_ concentration was predicted to reach above 3000 ppmv with the car ventilation fan off and the vehicle at rest carrying two passengers for more than 10 min [24]. This prediction is quite comparable to the idle engine condition from this study (mean 3096 ppmv), although in our tests, the car ventilation was mostly at recirculation mode, not completely turned off. In this type of situation, body odor accumulation may be a problem in the cabin. If fresh air with normal CO_2_ level of about 400 ppm is introduced into the car through the ventilation system (e.g., fresh air mode), the CO_2_ level accumulated from human source can be minimized. As seen in our data, some of the vehicles had a much higher CO_2_ level than that predicted by the human emission model, which may indicate permeation of a high level of CO_2_ into the cabin, such as, from polluted outdoor air and exhaust gas through leakage of ducting or piping.

According to the Occupational Safety and Health Administration (OSHA) (Washington, DC, USA) and the American Conference of Governmental Industrial Hygienists (ACGIH), the permissible occupational exposure limit for an 8-h workday is 5000 ppmv based on occupational health protection perspective [25,26]. In this study, we found 16% of the driving vehicles had CO_2_ levels above 5000 ppmv and the highest ones (four vehicles) could go up to a level that exceeded the measurement limit of the CO_2_ meter. These extremely high CO_2_ levels were beyond the concentration that was found in normal indoor settings and the occupational limit, although the overall exposure time was less than eight hours.

The CO_2_ levels measured in this study may not have posed a direct threat to the physical health of the commuters, but these measurements can cause dizziness and result in drowsy driving (e.g., >2000 ppm of CO_2_) [27], as well as lower the overall decision-making performance (studies conducted in a controlled environmental chamber with 1000 and 2500 ppm of CO_2_) [28]. Drivers need to be alert to different situations and to make decisions accordingly during driving. Thus, the high CO_2_ in this environment may constitute a potential risk to the commuters and road-safety.

### 4.2. Idling Engine: Everyone Pays

In Hong Kong, it is common for drivers to switch on the vehicle engine to enjoy the comfort of air-conditioning and reduce the air intake from the roadside polluted air while waiting for fellow passengers. This is particularly popular in school zones and shopping streets. Therefore, in 2011 the legislation council passed the Motor Vehicle Idling Ordinance to tackle this problem. The public education of the statutory ban against idling of motor vehicle engines focuses on the nuisance and harmful effects of car exhaust emission for pedestrians and shops along the roadside. Nevertheless, the in-cabin air quality while the vehicle is stationary with the engine running has not been sufficiently addressed. This study showed that, actually, everyone pays. Alarming levels of TVOCs can accumulate in the cabin during engine idling.

Compounds, such as benzene, toluene, ethylbenzene, xylene, etc. are known as vehicle exhaust gases. These compounds are emitted during fuel combustion in car engines or refueling and can penetrate into the interior of the cabin by natural circulation of air or suction through the ventilation system [15,17,20,29,30]. The concentrations of 10 major VOCs in static vehicles were significantly higher than in vehicles during dynamic conditions [31]. In addition, the VOCs concentration increases with a rise in temperature; when the temperature increased from 24 to 29 °C in vehicle cabins, a 28.8% increase in benzene concentration followed [32]. Furthermore, an increase in temperature from 24 °C to 35 °C resulted in a corresponding 1.5-fold increase in in-cabin formaldehyde concentration [33]. In summer time in Hong Kong, outdoor temperature can rise to above 30 °C regularly. With the additional heat generated from the car engine, even higher TVOCs emission and permeation into the cabin is possible.

To further illustrate the implications of our findings, assuming that a commuter stays in the cabin with >4000 ppbv of TVOCs (the highest concentration measured in our study) for 1 h, which is equivalent to the total TVOCs exposure as if working in a “not Good IAQ” office (with TVOCs level 262 ppbv) for over 15 h.

### 4.3. Improving In-Cabin Air Quality

The HKEPD recommends several measures for commuters to improve in-cabin air quality. Several points are relevant to this study and are discussed to highlight the current situation and the aspects that can be improved.

Firstly, it is recommended to open the fresh air vents or windows to introduce fresh air to the cabin, but close them when passing through polluted areas, such as tunnels or congested areas. In this study, we found that a majority of the commuters are aware of closing windows and operating the ventilation vents to the recirculation mode to reduce air pollutants entering the cabin, but none of them opened windows or switched the ventilation system to the fresh air mode during their journey. Most commuters (78%) rated the outside pollution level as “average to poor”. The commuters’ perception of the outside environment is likely to contribute to this behavior. In this study, PM_2.5_ levels decreased in the cabin over time irrespective of the ventilation modes but the level of PM_0.3_ was unaffected. However, Zhu et al. [3] reported that a vehicle’s car filter could help in reducing ultrafine particles (diameters less than 100 nm) by 20 to 50%, depending on the particle size. The lowest ultrafine particles concentration was obtained when using the recirculation mode of ventilation due to least air exchange between in-cabin and outdoors. In addition, they estimated that for an hour daily commute exposure, the in-cabin environment contributes approximately 10–50% of the daily exposure to ultrafine particles from traffic [3].

The in-cabin PM_2.5_ was significantly higher during driving than engine idling. This may suggest that PM_2.5_ is originated from the outdoor air. Previous studies showed that opening windows allows for outdoor air to penetrate the cabin, and PMs, in addition to other pollutants, tend to be concentrated, in particular during traffic congestion [34,35,36]. This study demonstrated that closing windows could help to lower the PM levels. The World Health Organization (WHO) advices that the lowest possible concentrations of PMs should be achieved [37]. Additionally, the EUROPART, European interdisciplinary group of researchers concluded that “there is inadequate scientific evidence for establishing limit values or guidelines for particulate mass or number concentrations” in the case of indoor particles [38]. Although the HKEPD IAQ guidelines do not include the PM_2.5_, a PM_2.5_ level of 24 µg m^−3^ was associated with pathophysiological complications that were shown in some studies [11]. Exposure to PM_2.5_ from traffic also affected the heart-rate variability, thrombosis, and inflammation [12]. Closing windows and switching the ventilation mode to recirculation can help to reduce PM levels, but these conditions could worsen the internal source and accumulation of VOCs, NO_2_, and CO_2_, because of the limited fresh air intake to dilute the pollutants [10,39].

Secondly, when servicing the vehicle in garage, the HKEPD suggests that people should request cleaning and replacing air filters and check any leakage of ducting and piping as exhaust gas can permeate into the cabin if leakage occurs [17,34]. In this study, our participants had little awareness of air filter maintenance. Moreover, with the very high CO_2_ and TVOCs levels in some vehicles, it is likely that the leakage of ducting and piping is quite possible, and in some worse cases, the pollutant levels were exorbitant.

Other tips include keeping the vehicle compartment clean and dry and removing moldy carpets to reduce microbial contamination. In this study, both airborne bacterial and fungal levels were low and no visible mold was observed in the cabin. However, if the microbial load is high, then a reduction may be achieved by replacing the car filter. A study showed that the microbial load was influenced by the filter and a 57% decrease in bacterial counts was observed, owing to changing the old filter [40].

For new vehicles, extra relief is proposed through ventilation of the cabin as much as practically possible to release VOCs from new fittings. Previous studies have suggested a decreasing trend of TVOCs with car age in the first 3–5 years [8,41,42]. In light of the fact that over 70% of our tested vehicles were more than three years old, the likelihood of TVOCs stemming from car fittings is relatively insignificant compared to other potential sources of VOCs.

Lastly, as engine idling could significantly deteriorate the in-cabin air quality within a short period of time (less than 10 min as shown in our study), commuters should minimize the time of engine idling.

## 5. Conclusions

This study gives some insights into the current state of in-cabin air quality in air-conditioned private vehicles in Hong Kong during driving and engine idling. The potential health hazards that are present in the vehicle and the implication of this study to the local recommendation to improve in-vehicle air quality were discussed. The concern of outdoor air pollution may lead to a regular enclosed cabin and increase commuter’s exposure to other pollutants that were generated by engine combustion or other internal sources. Although the time we spend in vehicles may be brief, the potential high pollutant exposure should not be overlooked.

## Figures and Tables

**Table 1:** Indoor air quality objectives in office buildings and public places in Hong Kong [21].

IAQ Parameters	Unit	8-h Average	
		Excellent class	Good class
Carbon dioxide (CO_2_)	ppmv	<800	<1000
Carbon monoxide (CO)	ppmv	<1.7	<8.7
Total volatile organic compounds (TVOCs)	ppbv	<87	<261
Airborne bacteria	CFUm^−3^	<500	<1000

**Table 2:** Questionnaire results regarding the vehicles and the driver perception of the outdoor and in-cabin air quality.

Questions	Response				
Car age	0–2 years 27.5%	3–5 years 31.4%	6–10 years 27.5%	10–18 years 13.6%	
Car mileage	0–30,000 km 31.4%	30,000–60,000 km 19.6%	60,000–90,000 km 25.5%	90,000–120,000 km 19.6%	No response 3.9%
Car usage	3–4 days per week 37.2%	5–6 days per week 37.2%	7 days per week 25.6%		
Frequency of cleaning	Every 1–2 weeks 21.6%	Every 1–2 months 33.2%	Every 1–2 years 21.6%	Never/Rarely 13.8%	Irregular 9.8%
Filter maintenance	Every 6 months 3.7%	Every 10,000 km 3.9%	Twice yearly 9.8%	Never 72.6%	
Filter types	Don’t know 96.1%	Original 3.9%			
Suspected agents that could contribute to in-cabin TVOCs	No 68.6%	Plastic toys 2.0%	Fragrance 17.6%	Alcohol gel 3.9%	Do not know 7.9%
Air conditioner usage	All year 64.7%	Summer only 31.4%	Irregular 3.9%		
Ventilation mode	Fresh air 19.6%	Recirculation 72.5%	Irregular 7.9%		
Perceived pollution level along the travel route	Low 21.6%	High 21.6%	Average 56.8%		
Any odors in cabin	No 70.6%	Yes 29.4%			
Smoking in cabin	No 94.1%	Yes 5.9%			
